# Reading Comprehension Assessment through Retelling: Performance Profiles of Children with Dyslexia and Language-Based Learning Disability

**DOI:** 10.3389/fpsyg.2016.00787

**Published:** 2016-06-01

**Authors:** Adriana de S. B. Kida, Clara R. B. de Ávila, Simone A. Capellini

**Affiliations:** ^1^Department of Speech-Language Pathology and Audiology, Universidade Estadual Paulista “Júlio de Mesquita Filho”Marília, Brazil; ^2^Department of Speech-Language Pathology and Audiology, Universidade Federal de São PauloSão Paulo, Brazil

**Keywords:** reading comprehension, retelling, simple view of reading, dyslexia, recall pattern

## Abstract

**Purpose:** To study reading comprehension performance profiles of children with dyslexia as well as language-based learning disability (LBLD) by means of retelling tasks.

**Method:** One hundred and five children from 2nd to 5th grades of elementary school were gathered into six groups: Dyslexia group (D; *n* = 19), language-based learning disability group (LBLD; *n* = 16); their respective control groups paired according to different variables – age, gender, grade and school system (public or private; D-control and LBLD-control); and other control groups paired according to different reading accuracy (D-accuracy; LBLD-accuracy). All of the children read an expository text and orally retold the story as they understood it. The analysis quantified propositions (main ideas and details) and retold links. A retelling reference standard (3–0) was also established from the best to the worst performance. We compared both clinical groups (D and LBLD) with their respective control groups by means of Mann–Whitney tests.

**Results:** D showed the same total of propositions, links and reference standards as D-control, but performed better than D-accuracy in macro structural (total of links) and super structural (retelling reference standard) measures. Results suggest that dyslexic children are able to use their linguistic competence and their own background knowledge to minimize the effects of their decoding deficit, especially at the highest text processing levels. LBLD performed worse than LBLD-control in all of the retelling measures and LBLD showed worse performance than LBLD-accuracy in the total retold links and retelling reference standard.

Those results suggest that both decoding and linguistic difficulties affect reading comprehension. Moreover, the linguistic deficits presented by LBLD students do not allow these pupils to perform as competently in terms of text comprehension as the children with dyslexia do. Thus, failure in the macro and super-structural information processing of the expository text were evidenced.

**Conclusion:** Each clinical group showed a different retelling profile. Such findings support the view that there are differences between these two clinical populations in the non-phonological dimensions of language.

## Introduction

Reading comprehension assessment by retelling a previously read text enables direct access to the expression of mental representation built by the reader (Leslie and Caldwell, 2009; Reed and Vaughn, 2012) without any interference or facilitation. It also evidences one’s competence to both identify relevant information of the previously read text and integrate these ideas into a cohesive and coherent global scheme (Orrantia et al., 1990; León, 1991; García Madruga et al., 1996). Retelling, allows for an outlining of different levels of comprehension, these being the reading processing product resultant from the micro, macro and super-structures (Squires et al., 2014; Kida et al., 2015). The identification of the total retold ideas is a direct measure of how the reader operates with the basic units of the text – the propositions – and it reflects his ability to keep them in mind (Orrantia et al., 1990; Carlisle, 1999). These abilities are intricately connected to the micro-structural comprehension, therefore, to the local information processing level (Kintsch and Keenan, 1973; Frazier and Fodor, 1978). The total retold ideas, mainly when considering the importance of each one to the textual chain (main ideas or details), gives clues of the reader’s abilities to choose relevant information from the text and to start the global processing of the text macro-structure. After choosing, generalizing and leaving ideas out, skills involved in this process, the reader starts establishing connections between each piece of information, in other words, the links. Retelling also provides the product of global processing at a macro-structural level. Such product may be assessed by the total links made between the retold ideas, and by the retelling reference standard (Squires et al., 2014). The measurement provided by the retelling standard takes into account the analysis of the set of retold links considering its relevance for the text central chain. For that reason, such measurement helps determine the comprehension level achieved by the reader and, therefore, the way he reaches the text super-structure. The reference standard of the retelling of expository texts evaluates the way the reader organizes his ideas toward an established central goal (Bustos Ibarra, 2009; Squires et al., 2014), guided by his knowledge of the text structure (Richgels et al., 1987; Roller, 1990).

Hence, the set of retelling measurements – total retold ideas, total links and the retelling reference standard – may provide hints of how the reader builds his understanding of the text and at what level of processing difficulties remain when reading comprehension does not take place (Owens et al., 1979; Orrantia et al., 1990; Bernhardt, 1991).

Reading comprehension difficulties result from varied types of reading deficits, identified by the reader’s performance in automatic recognition of written words and/or in oral comprehension. Three clinical groups are known: (1) Readers with specific reading comprehension deficits; (2) Readers with specific decoding deficits – dyslexia; (3) Readers with both deficits – known as language-based learning disability (LBLD) deficits (Catts et al., 2003) or also as mixed deficits (Catts et al., 2005b; Cain and Oakhill, 2006). The differences between the latter two clinical groups are primarily in non-phonological language dimensions. Children with LBLD show, besides a phonological processing impairment, a typical dyslexia symptom, significant deficits of oral comprehension (Aaron, 1991; Catts and Kahmi, 2005) with hindrance to vocabulary, morph syntax, and text structural processing, even when their non-verbal abilities are preserved. These linguistic deficits are, therefore, broad and, they interact directly with reading competences, resulting in different manifestations, thus making reading problems in the LBLD group more evident and, equally broad (Catts, 1993; Catts et al., 1997, 2005a; Bishop and Snowling, 2004).

Although children with dyslexia and with LBLD knowingly show difficulties of very different nature, it is acknowledged that reading comprehension can be impaired in both cases. Children with dyslexia may present reading comprehension difficulties influenced by their decoding deficits, despite their good oral comprehension. Their slow and inaccurate word recognition may limit sentence and text processing speed, thus resulting in comprehension impairments (LaBerge and Samuels, 1974; Perfetti, 1985; Shankweiler et al., 1999). Pupils with learning disabilities, in turn, show deficit in reading comprehension as a consequence of poor decoding abilities and of more general language deficit (Stanovich and Siegel, 1994; Ellis et al., 1996; Aaron et al., 1999).

Literature has not yet shown if retelling allows for an identification of different performance profiles in reading comprehension among the different cases of reading impairment. The presence of heterogeneous groups (poor readers, reading disabled children, and children with learning disabilities) in the searched studies does not help understand the effects of the deficits in different competences (decoding and language) upon reading comprehension.

Studies showed that these children with learning difficulties, designated to the sample upon their teachers’ recommendation or chosen according to the identification of deficits in their reading performance on specific evaluation tests, significantly retold less pieces of information. Furthermore, they showed worse oral discourse management of the retelling due to their difficulties in adequately choosing main ideas rather than details (Williams, 1991; Curran et al., 1996; Carlisle, 1999; Reed and Vaughn, 2012). Even in studies that encouraged retelling, requesting children to add more pieces of information at the end of their narrative, showed that there was no retelling expansion. The retelling of children with academic learning difficulties showed no expansion of number of ideas (Bridge and Tierney, 1981; Zinar, 1990; Reed and Vaughn, 2012), as opposed to the performance of good readers.

When considering such particular nature of deficits that affect children with dyslexia and children with LBLD, it is expected the investigated clinical groups (D, LBLD) to show distinct performance profiles in reading comprehension at different levels of text processing. The chances of the retelling identify the quality of mental representation generated by the text, by means of measuring the total of retold ideas (main and details), links and the reference standard that each one of those clinical groups grasped, would help understand which strategies of base-text construction could be impaired. Then, it is necessary to compare the performance of each clinical group with the performance of its pairs of the same age, gender, school grade, as well as with pupils of the same reading accuracy level.

Cain et al. (2000) proposed an experimental design based on the use of comparison by pairing levels of competence. The idea of this design is to compare an interest group with pairs of the same age and school grade as a means of controlling the effect of variables, such as language development and schooling, upon reading performance. Moreover, comparing accuracy competence groups helps not only to demonstrate if the performance of a clinical group is below its pairs of the same level of development and schooling, but also to advance in understanding the cause of reading difficulties (Frith and Snowling, 1983; Siegel and Ryan, 1988). The comparison between comprehension competence of a clinical group and younger pupils of equivalent decoding competence may help determine what is the most probable explanation for the relationship between both competences: is reading comprehension proficiency due to linguistic development or to decoding competence? If the group without complaints of reading difficulties, paired by age, gender and schooling shows better reading comprehension competence, we may suppose that decoding competence may interfere in comprehension access, once this competence distinguishes these groups. However, if the performance of pupils with difficulties is better than pupils paired by level of accuracy, for example, we may presume that the schooling and/or the language development, a distinguishing factor between the groups, may favor comprehension performance. At last, if the performance of pupils with difficulties is worse than the group paired by level of accuracy, it is presumable that, once decoding is controlled, the deficits in language competence may interfere in the performance of pupils with difficulties.

This experimental design is intended to help clarify the nature of reading comprehension difficulties faced by children with dyslexia and by children with language-based learning disabilities. Findings are intended to help in the comprehension of the necessary supports and to facilitate the planning of required intervention by each of these groups of readers.

### Purpose

This study aimed at characterizing oral retelling profiles made by children with dyslexia and LBLD, after reading an expository text.

First, we intended to identify the points of comprehension breakdown at different text processing levels (micro structure, macro structure, and super structure), as well as to measure the effects of deficits based on the expected performance according to age and schooling. For such, clinical groups were compared with its controls of the same level of development and schooling.

Afterward, we meant to understand those comprehension breakdowns (micro-structure, macro-structure, and super-structure) based on the language competence shown by the clinical groups. For such, the decoding variable was controlled through pair ups according to accuracy level.

Thus, we tried to answer the following research questions starting from the designed hypotheses.

(a) Do children with dyslexia, who present restricted decoding difficulties, necessarily show reading comprehension impairments? At what text processing level do decoding difficulties interfere to the point of impairing reading comprehension? Can skilful language competence compensate for decoding deficits? Also, at which level processing of textual information could language favor reading comprehension?
  In the light of those questions, we adopted the following hypotheses:
  - Children with dyslexia (D) will show reading comprehension difficulties when compared with the group paired by two development parameters – age and schooling (school grade and school system) – (D-control). By that, it may be possible to assume that the presented decoding deficits may generate negative effects on reading comprehension.
  - Children with dyslexia will show better performance when compared with the reading accuracy level group (D-accuracy). By that, it may be possible to assume that children with dyslexia are able to employ their most skilful language competences and to make use of their knowledge, built throughout their longer schooling period, in favor of reading comprehension performance. For that reason, they will show better chances of processing information than younger children with the same decoding competences.
(b) Will children with LBLD, who show both decoding and language competence difficulties, present specific alterations at certain levels of reading comprehension, or a broader compromised profile in processing textual information? At which levels of processing textual information may the deficits present in language interfere in reading comprehension activities?
  In turn, for children with LBLD, we proposed the following hypotheses:
  - Children with LBLD will show difficulties in every level of processing of textual information when compared with the children of the same level of development, gender and schooling (LBLD-control).
  - Children with LBLD will show similar or worse performance when compared with pupils of the same reading accuracy level (LBLD-accuracy). That result will allow us to assume that their language competences will not be enough to compensate for their decoding difficulties, making their reading comprehension performance similar or even worse than this control group. Such result will depend on the extent of the effect of linguistic competences this population has.

## Materials and Methods

The sample consisted of 105 students, native Brazilian Portuguese speakers from the 2nd to the 5th grades of Elementary School, without complaints nor indicators of hearing or visual impairment, neurological, behavioral or cognitive disability. They comprised six groups: (a) D: 19 children (53% male, average age: 127 months, *SD* = 16.1) with clinical developmental dyslexia diagnostic; (b) D-control: 19 children (53% male, average age: 123 months, *SD* = 15) without complaints of reading difficulties. This group was paired up with D according to age, gender, school system and schooling parameters; (c) D-accuracy: 19 children (47% male, average age: 123 months, *SD* = 2.1) paired up with D according to reading accuracy; (d) LBLD: 16 children (81% male, average age: 122.5 months, *SD* = 14.7) with clinical diagnostic of LBLD; (e) LBLD-control: 16 children (81% male, average age: 121 months, *SD* = 10.4). This group was paired with LBLD according to age, gender, school system and schooling; (f): LBLD-accuracy: 16 children (44% male, average age: 86.5 months, *SD* = 3.9) paired with LBLD according to reading accuracy.

Clinical group participants (D abd LBLD) were recruited through cross-disciplinary diagnostics (neurologist, neuro-psychologist, psycho-pedagogue, and speech therapist) carried out at Laboratório de Investigação dos Desvios de Aprendizagem do Centro de Estudos da Educação e da Saúde da Faculdade de Filosofia e Ciências – CEES/FFC/UNESP Marília – SP (Learning Deviation Investigative Laboratory of the Centre of Health and Education Studies of the Philosophy and Science College – CEES/FFC/UNESP Marília – SP) and at Laboratório dos Desvios de Aprendizagem do Hospital das Clínicas da Faculdade de Medicina – HC/FM/UNESP – Botucatu – SP (Learning Deviation Laboratory of the Clinics Hospital of Medicine College – HC/FM/UNESP – Botucatu – SP).

D pupils showed (1): an expected intelligence quotient (I.Q equal to or higher than 80) in psychological evaluation, (2) the presence of significant discrepancy between the verbal and the execution quotients, with differences leaning toward the execution IQ, with lowering score in the digit subtest and good performance at vocabulary and arithmetic subtests taken according to the expected values for the age at WISC-III (Wechsler, 2002); (3) low performance in the reading of both the isolated word task, according to parameters established for the standard test for the Brazilian school population (Stein, 1994) and the pseudo words (Arduini et al., 2006; Salgado and Capellini, 2008); (4) performance impairment of phonological short-term memory, according to the expected schooling parameters (Kessler, 1997; Tabaquim, 2008); (5) poor performance in the rapid serial naming task (Denckla and Rudel, 1974) according to parameters of the Brazilian school population (Simões, 2006); (6) performance impairment in the phonological awareness task, showing a lowering of more than 1.5 dp of the total score for the schooling (Capovilla and Capovilla, 1998).

Language-based learning disability participants met the following inclusion criteria (Puranik et al., 2006): (1) history of previous language impairment or academic difficulties in early school years; (2) intellectual quotient below average (minimum I.Q of 80 points) with the absence of discrepancy between the verbal and the execution intellectual quotient at the WISC-III psychological assessment (Wechsler, 2002); (3) the same or better performance than the percentile 25 (below average level) at the Raven’s Progressive Matrices, with schooling parameters taken into account (Raven et al., 1988); (4) good performance at the Wisconsin Card Sorting test classification, with schooling parameters taken into account (Heaton et al., 2005); (5) poor performance in the tasks of reading isolated words, according to the Brazilian schooling population parameters for writing and Arithmetic (Stein, 1994).

The control group participants were recruited in Elementary Schools of the same city. Besides meeting the recruitment criteria established for the whole sample, these children did not show history of speaking nor language impairment, of academic or reading difficulties, neither suggestive signals of sensory alterations, neurological and cognitive impairment, according to their teacher’s designation.

The study of pairing up clinical groups (D and LBLD) with their controls of the same age, gender, schooling and school system (D-control and LBLD-control) in relation to the age variable was carried out by means of the one-way analysis of variance (ANOVA One Way), making use of age (in months) as a dependent variable. Bonferroni Tests were performed in order to verify the existence of differences between the pairs of groups. Results showed that there were no differences between the groups [D and D-control: *F*(1,36) = 0.46, *p* = 0.500, η^2^ = 0.907; LBLD and LBLD-control: *F*(2,35) = 6.38, *p* = 0.500, η^2^ = 0.026].

Mann–Whitney test was used to assess the pairing up of clinical groups (D and LBLD) with their controls according to age and schooling (D-control and LBLD-control) and with their controls according to reading accuracy (D-accuracy and LBLD) for the decoding variable. Rate (number of words read per minute) and accuracy (number of correct words read per minute) were used as dependent variables and group as a fixed factor.

The comparison of the decoding variables (rate and accuracy) resultant from the single item task (Pinheiro, 2011) showed that the clinical groups (D and LBLD) presented lower figures when compared with their control pairs according to age, gender, and schooling (**Table 1**). In turn, the comparison between the clinical groups and their controls, paired according to their level of reading, showed similar figures (**Table 2**). Results proved the decision of pairing up these groups appropriate, considering the adoption of the experimental design.

**Table 1:** Study of the sample pair up based on reading fluency variables – Comparison between clinical groups and control groups according to age, gender and schooling.

Variables		Groups			
	
		D	D-control	LBLD	LBLD-control
		(*N* = 19)	(*N* = 19)	(*N* = 16)	(*N* = 16)
Reading rate	Mean	25.9	56.5	23,1	63,1
	*SD*	11.2	11.3	8,4	19,1
	*U*	9.50		11.00	
	*P*-value	*p* < 0.001		*p* < 0.001	
	Results	D < D-control		LBLD < LBLD-control	

Reading accuracy	Mean	11.9	49.2	8,5	54,6
	*SD*	9.1	12.2	5,5	6,3
	Z	9.00		6.50	
	*P*-value	*p* < 0.001		*p* < 0.001	
	Results	D < D-control		LBLD < LBLD-control	


*Mann–Whitney test. D, Dyslexia Group; D-control, Dyslexia control group by age, gender, and grade; LBLD, Language-based learning disability group; LBLD-control, Language-based learning disability control group by age, gender and grade. Significant at *p* < 0.05.*

**Table 2:** Pairing up data between clinical groups and their control groups paired according to reading accuracy upon reading fluency variables.

	Variable	Groups			
	
		D	D-accuracy	LBLD	LBLD-accuracy
		(*N* = 19)	(*N* = 19)	(*N* = 16)	(*N* = 16)
Rate	Mean	25.9	24.0	23.1	27.6
	*SD*	11.2	8.12	8.4	15.2
	U	153.50		119.50	
	*P*-value	0.219		0.234	
	Results	D = D-accuracy		LBLD = LBLD-accuracy	

Accuracy	Mean	11.9	11.6	8.7	9.1
	*SD*	9.1	10.3	5.5	6.3
	U	170.5		124.50	
	*P*-value	0.625		0.237	
	Results	D = D-accuracy		LBLD = LBLD-accuracy	


*Mann–Whitney test. D, Dyslexia Group; D-accuracy, Dyslexia control group by reading accuracy; LBLD, Language-based learning disability group; LBLD-accuracy, Language-based learning disability control group by reading accuracy. Significant at *p* < 0.05.*

### Procedures

#### Protocol of Retelling after Reading

Four expository texts were carefully written about subjects that were not part of the private and public school programs, neither in their previous grades nor in the intended evaluated grade. Such criterion aimed at minimizing the effects of the participants’ previous knowledge of the reading comprehension assessment.

The texts proved appropriate for each school grade. A previous study revealed that the texts were appropriate for: readability (analyzed through the Flesch Index), syntax complexity (Indexes: number of words and sentences of the texts, number of sentences within paragraphs, occurrence of content words, pronouns per syntagma, and number of linkers) and vocabulary complexity (Type/Token Index), attested parameters that interfere in comprehension (Aluísio et al., 2008; McNamara et al., 2010; McNamara et al., 2012). All of these measurements were achieved through the *CohMetrix-Port* computerized tool (Scarton and Aluísio, 2010) and were compared with the expected parameters for texts of each researched school grade (Kida, 2015). These reference figures were established based on the analysis of 15 schoolbook collections of the Portuguese Language (total of 918 texts), approved by the Plano Nacional do Livro Didático (Schoolbook National Plan) – PNLD 2013 (Brasil, 2012), intended to the teaching of pupils from the 2nd to the 5th grades of Elementary School. Changes were made in the texts once comparisons showed inadequacies on a certain assessed parameter. Such changes assured that the final texts had the desirable syntax complexity and readability for each school grade.

The analysis of the retellings was carefully sifted through three assessors based on the propositions of each text. Such analyses were compared and the propositions classified, under consensus, as main ideas and details. For such, the importance of each proposition for the main chain of the text and the nature of the conveyed information (explicit or implicit) were considered. The identified propositions composed the screening and allowed for the identification of total retold ideas, a parameter used to assess the processing of text micro-structure (Sánchez, 2002; Bustos Ibarra, 2009).

Assessors also identified the existing links in the text (Sánchez, 2002; Bustos Ibarra, 2009). Links were understood as causal connection between main ideas, between main ideas and details or between details. Every identified link composed the screening and allowed for the assessment of the quality of the global processing of textual macro-structure.

Another adopted parameter was the retelling standard reference, established according to the following criteria (Coté et al., 1998; Gonzales, 2008; Bustos Ibarra, 2009): Standard 3–presence of all the links between main ideas, together with, at least, one link between main ideas and details; Standard 2–presence of links between main ideas, without the presence of links between main ideas and details; Standard 1–presence of, at least, one link, no matter its classification; Standard 0–absence of causal connections of any type. These standards were established to reflect different ways of organizing memorized ideas toward the central objective of the text, thus allowing for the observation of the super-structural processing of the text by the reader (Bustos Ibarra, 2009).

#### Data Collection Procedures

The assessed children were instructed to read the text that was assigned for their school grade the habitual way they read for comprehension (oral or silently). No time limit was defined. They retold the previously read information when they considered they were ready. Their retellings were recorded for later transcription and analysis.

#### Procedures of Data Analysis

##### Procedures of analysis of inter-rater agreement

Four speech therapists were trained to analyze the retellings. After training, transcriptions of 200 retellings (50 of each school grade) from previous researches were used and helped achieve the inter-rater agreement. Such measure intended to guarantee reliability. To estimate the Reliability Indexes, the Kappa inter-observer agreement measurement was used, establishing 0.70 as a minimum level (Urbina, 2007). The resulting indexes for each one of the texts are in **Table 3**.

**Table 3:** Inter-rater Agreement index for variables of analysis of retelling after reading expository texts assigned to each school grade.

Retelling analysis variables	Expository texts			
	
	2nd grade	3rd grade	4th grade	5th grade
Tot_ID	0.730	0.817	0.704	0.703
Tot_Links	0.928	0.898	0.752	0.801
RRS	0.862	0.961	0.760	0.850


*Kappa Test. Tot_ID, total of ideas retold; Tot_links, total of links retold; RRS, retelling reference standard. Cut-Off: 0.70.*

##### Procedures of retelling analysis

Once the inter-observer agreement was proven, the participants’ retelling transcriptions were distributed among the four coders. It was a blind assessment as for age, gender, and schooling of participants, as well as their groups.

Coders identified each one of the ideas and retold links and scored one point for each piece of information identified. Then, they summed the total ideas and links. Eventually, they identified the retelling standard with its respective scorings.

Gross results of total ideas and links were converted based on *z*-scores, as lower, average, and upper levels, to which 0, 1, and 2 scores were, respectively, assigned (Kida, 2015). The data resulting from the analysis of each retelling were tabulated and submitted to statistical analysis.

### Data Analyses

The normality test indicated the presence of non-normal distribution for the analysis variables. The comparative analysis of performance of clinical groups with their respective controls – comparison of independent groups, two by two – was carried out through the Mann–Whitney test for the total ideas, total links and retelling standard. The significance level adopted was *p* < 0.05.

The extent of the effect size for the Mann–Whitney test was assessed by approximation of the distributions of test statistics for the Z distribution, once this is a non-parametric test. Thus, the following calculation was made: *r* = Z/√N. The analysis criteria for effect size (proposed upon the Cohen *r*) and adopted for this study were: great effect = 0.5; average effect = 0.3; low effect = 0.1 (Fritz et al., 2012).

The statistical pack IBM SPSS Statistics – version 22 (pt) – was used in the analysis mentioned above.

## Results

### Study Results of the Retelling Profile of Children with Developmental Dyslexia

**Table 4** shows that children with dyslexia (D) presented performance similar to their pairs of the same age, gender and schooling (D-control) for the variables of total ideas and of retold links, as well as of retelling standard after reading.

**Table 4:** Reading comprehension performance of D and D-control in retelling task.

		Group		Mann–Whitney test	Result
				
		D	D-control		
Tot_ID	Mean	0.63	1.00	*p* = 0.354	D = D-control
	*SD*	0.495	0.577		

Tot_Links	Mean	0.263	0.895	*p* = 0.402	D = D-control
	*SD*	0.452	0.809		

RRS	Mean	0.263	0.895	*p* = 0.258	D = D-control
	*SD*	0.452	0.567		


*Tot_ID, total of ideas retold; Tot_links, total of links retold; RRS, retelling reference standard; SD, standard deviation; D, Dyslexia Group; D-control, Control group by age, sex and grade.*

The comparative investigation of total retold ideas showed that D retold less central ideas, significantly differing from its pairs of the same age and schooling (D: 0.42/D-control: 1.11; *U* = 150.00, *p* = 0.004, *r* = 0.542, I.C. 95%: lower limit = -0.7, upper limit = -1.09). However, D and D-control performances were similar when compared with the total retold details (D: 0.58/ D-control = 0.89; *U* = 21.00, *p* = 0.154).

Comparisons between children with dyslexia (D) and their pairs of the same level of reading accuracy (D-accuracy) showed that children with dyslexia presented better performance evidenced by the greater number of retold links (*r* = -0.1286, I.C. 95%: lower limit = -0.905, upper limit = -0.1667) and by the retelling score (*r* = 0.1286, I.C. 95%: lower limit = -0.905, upper limit = -0.1664), as observable in **Table 5**.

**Table 5:** Reading comprehension performance of D and D-accuracy in retelling task.

		Group		Mann–Whitney test	Result
				
		D	D-accuracy		
Tot_ID	Mean	0.632	0.315	*p* = 0.488	D = D-accuracy
	*SD*	0.495	0.477		

Tot_Links	Mean	0.263	0.105	*p* = 0.005^∗∗∗^	D > D-accuracy
	*SD*	0.452	0.315		

RRS	Mean	0.263	0.105	*p* = 0.005^∗∗∗^	D > D-accuracy
	*SD*	0.452	0.3153		


*Tot_ID, total of ideas retold; Tot_links, total of links retold; RRS, retelling reference standard; SD, standard deviation; D, Dyslexia Group; D-accuracy, control group by reading accuracy; significant at *p* < 0.05, ^∗^*p* < 0.05, ^∗∗^*p* < 0.01, ^∗∗∗^*p* < 0.001.*

The comparative investigation of the total retold ideas showed that dyslexic children (D) presented similar performance to the observed in children with the same level of accuracy (D-accuracy; Main ideas – D: 0.42/D-accuracy: 0.11; *p* = 0.096; Details: D: 0.58/D-accuracy: 0.26; *p* = 0.096).

### Study Results of the Retelling Profile of Children Diagnosed with Language-Based Learning Disability (LBLD)

**Table 6** shows the results of the comparison between the performance of children with LBLD and their pairs of the same age, gender, and schooling (LBLD-control) as for the total ideas, links and retelling standard. LBLD showed significantly poorer performance than LBLD-control in every measure of retelling (Total retold ideas: *r* = 0.1286, I.C. 95%: lower limit = -0.0872, upper limit = 0.1770; Total retold links: *r* = 0.0952, I.C. 95%: lower limit = -0.0431, upper limit = 0.1473; RRS: *r* = 0.0168, I.C. 95%: lower limit = -0.0076, upper limit = 0.0262). The comparative analysis showed that children from the LBLD group presented similar performance both in main ideas and details (Main ideas: LBLD: 0.25/LBLD-control: 1.06, *U* = 44.00, *p* = 0.268, *r* = 0.0169, I.C. 95%: lower limit = -0.0713, upper limit = 0.1050; Details: LBLD: 0.25/LBLD-control: 0.87, *U* = 44.00, *p* = 0.236, *r* = 0.0233, I.C. 95%: lower limit = -0.0102, upper limit = 0.0364).

**Table 6:** Reading comprehension performance of LBLD and LBLD-control in retelling task.

		Group		Mann–Whitney test	Result
				
		LBLD	LBLD-control		
Tot_ID	Mean	0.437	1.187	*p* = 0.002^∗∗∗^	LBLD < LBLD-control
	*SD*	0.512	0.403		

Tot_Links	Mean	0.125	1.125	*p* = 0.003^∗∗∗^	LBLD < LBLD-control
	*SD*	0.341	0.806		

RRS	Mean	0.062	0.875	*p* = 0.047^∗∗^	LBLD < LBLD-control
	*SD*	0.250	0.719		


*Tot_ID, total of ideas retold; Tot_links, total of links retold; RRS, retelling reference standard; SD, standard deviation; LBLD, Language-based learning disability group; LBLD-control, control group by age, sex, and grade; significant at *p* < 0.05; ^∗^*p* < 0.05, ^∗∗^*p* < 0.01, ^∗∗∗^*p* < 0.001.*

The performance of children with LBLD and of their pairs of the same level of accuracy (LBLD-accuracy) showed to be similar in every variable of retelling performance, as observable in **Table 7**.

**Table 7:** Reading comprehension performance of LBLD and LBLD-accuracy in retelling task.

		Group		Mann–Whitney test	Result
				
		LBLD	LBLD-accuracy		
Tot_ID	Mean	0.437	0.625	*p* = 0.239	LBLD = LBLD-accuracy
	*SD*	0.512	0.500		

Tot_links	Mean	0.125	0.375	*p* < 0.001	LBLD < LBLD-accuracy
	*SD*	0.341	0.619		

RRS	Mean	0.006	0.187	*p* < 0.001	LBLD < LBLD-accuracy
	*SD*	0.250	0.403		


*Tot_ID, total of ideas retold; Tot_links, total of links retold; RRS, retelling reference standard; SD, standard deviation; LBLD, Language-based learning disability group; LBLD-accuracy, control group by reading accuracy; significant at *p* < 0.05.*

As for the retold ideas, however, the typical children paired according to level of accuracy (LBLD-accuracy) retold as much main ideas and details as the children with LBLD (Main ideas: LBLD: 0.25/LBLD-accuracy: 0.56, *U* = -16.00, *p* = 0.346; details: LBLD: 0.25/ LBLD-Ac: 0.75, *U* = -44.00, *p* = 0.579).

## Discussion

### Profile of Retelling of Children with Developmental Dyslexia

The comparison between the performances of children with developmental dyslexia and typical children of the same age, gender, and schooling showed that decoding problems did not interfere in the performance measured by the most general parameters of the retelling (total ideas and retold links, reference standard). These results oppose the initial hypotheses.

Puranik et al. (2008) also observed similar performance within pupils with developmental dyslexia and their controls of the same age and schooling assessed through a written retelling task. Both groups showed the same total retold ideas. The authors assign the good performance of the dyslexia group to the children’s skilful language competences, which allowed them to compensate for their reading decoding deficits.

However, even considering the fact that the total retold ideas did not affect group performances at the present study, children with important reading decoding impairment showed poorer performance as for the number of main ideas retold. Such result shows that children with dyslexia were less efficient than typical readers of the same age and schooling in identifying and choosing main ideas, an important competence for textual processing at its macro-structural level. These children’s worse performance in retelling main ideas suggests that decoding difficulty interfere in the use of macro-rules employed in the construction of the mental representation of the text (Kintsch, 1988, 1998), causing loss of recent information as well as difficulty in leaving out less relevant data (Weaver and Dickinson, 1979).

Similar results were found by Snyder and Downey (1991) and Nascimento et al. (2011). These finding showed that retelling after reading with a smaller number of main ideas happened among readers with poor performance in reading fluency, evidenced by the low rate and accuracy estimates in tasks of reading recognition of isolated words. However, in this study, decoding automaticity failures and the effects they implied to the processing of syntactic information impaired comprehension of the connections of ideas (Snyder and Downey, 1991; Nascimento et al., 2011). The absence of readiness for information processing at a micro-structural level did not allow poor readers to draw their attention and their meta-cognitive resources to process high-level information (identification of the main subject of the text and recognition of the textual structure), primal in regulating macro-rule employment (Orrantia et al., 1990; Snyder and Downey, 1991; García Madruga et al., 1996; Nascimento et al., 2011). Inefficient use of macro-rules (choosing and leaving information out based on its importance for the chain of the text) determined the effects on the progressive construction of the mental representation during reading.

Overall, literature implies that competition between the decoding and the comprehension competences may impair access to the meaning of words and to the quality of syntactic processing (Vogel, 1975) and, consequently, interfere in the construction of a mental representation and/or the transference of information to the long-term memory (Shankweiler and Liberman, 1972; Juel, 1988; Shankweiler et al., 1999; Bowey, 2000). Furthermore, studies suggest that the natural limitation of the operational memory to manage information during reading at the presence of decoding problems compromises reading comprehension. Competition between decoding and comprehension by the operational memory builds a barrier that can restrict the use of language high-level processing systems, required by the global comprehension of the previously read content (Perfetti and Lesgold, 1977; Snyder and Downey, 1991).

Data analysis of the present study did not reveal, as expected, a worse performance of the group of children with developmental dyslexia for macro and super-structural measurements, in other words, for total links and retelling standard.

That way, although the decoding effects on reading comprehension are known, a theoretical view seeks to explain how underlying competence differences may influence reading at an interactive and compensatory perspective (Stanovich, 1980; Perfetti and Roth, 1981). Evidences that the nature of the parallel processing of reading may compensate for decoding difficulties have been broadly demonstrated. Children with dyslexia show better competence at using contextual facilitation than typical readers, because of the adequacy of their oral comprehension competence (Nation and Snowling, 1988). Attention to contextual information within texts frequently serve children with dyslexia to solve decoding ambiguities based on the collection of hints and the use of their previous knowledge, always through the action of their preserved cognitive and linguistic competences (Nation and Snowling, 1988).

A study carried out by Shankweiler et al. (1999) showed that pupils with reading decoding difficulties can compensate such difficulties with the use of their good linguistic competences through a “top-down” processing. But, contradicting the results of the present study Shankweiler et al. (1999) indicate that this compensatory competence is limited, viewing that it was not able to level the performance of dyslexic pupils to the one of typical readers. Lots of authors argue that the competence of compensating decoding impairments is more often observable in students with dyslexia who have been studying for a longer period of time, such as adolescents and adults (Campbell and Butterworth, 1985; Simmons and Singleton, 2000). At a younger age, compensation seems to contribute only to literal processing of information, not reaching interferential competences (Miller-Shaul, 2005).

Although compensation may be a hypothesis, data observation allows us to suppose that the similar performance of pupils with dyslexia and their pairs of the same age and schooling happened because of the low performance of the control group. When considering the control of the school system variable (private or public), impairment becomes evident as for the performance of reading comprehension also of readers taken as proficient by their teachers.

National assessment of reading comprehension in Brazilian Primary Schools and Middle School/Junior High (from the 1st to the 9th grades) shows that 45% of students with 4 years of schooling, after the beginning of the literacy process, present low reading comprehension levels (Brasil, 2014). Although they manage to deal with explicit information, to make connections between the text information or, to a certain extent, make use of their knowledge of the world, most of these students can only use these competences in simple texts (Bridon and Neitzel, 2014), below the expected level for their schooling.

Data collected from the control of the decoding effects on reading comprehension showed that the performance of children with dyslexia was significantly better, considering the number of retold links and the retelling standard achieved when compared with its pairs of the same level of accuracy. Data suggest that better language competence and the experience reached through a longer schooling period of children with dyslexia provide them with greater competence of connecting the processed ideas at a macro-structural level, as well as of incorporating them into a broader textual scheme.

These findings corroborate the hypothesis that decoding difficulties may be minimized when two linguistic competences are present. Thus, it may be assumed that the best language competences of children with dyslexia can be used in favor of a more efficient processing of the macro-structure of the text, having, therefore, greater competence in integrating main identified ideas (total links) and in its integration into a general textual scheme (reference standard of the retelling).

Orrantia et al. (1990) defend the idea that competent readers use varied cognitive operations to achieve global meaning (Meyer, 1984; Kintsch, 1998): choice, generalization, integration, and suppression of propositions. However, when these strategies come together with recognition of the global structure of the text, organization and integration of the propositions chosen at a coherent global scheme are even more efficient. These high-level competences are connected both to a good language development, for integration and choice, and to the reading experience, for recognition of the global structure of the text (García Madruga et al., 1996). That way, although children with dyslexia presented the same possibilities of identifying and retelling main ideas than pupils of the same level of reading, their greater reading experience has probably allowed them to transfer such gains to the processing of certain macro and super-structural levels of the text, results corroborated by Weaver and Dickinson (1979) and Kornev and Balciuniene (2014).

### Profile of the Retelling of Children Diagnosed with Language-Based Learning Disability

Children with LBLD showed important difficulties in reading comprehension, presenting poorer performance at all text processing levels when compared with their pairs of the same age and schooling. This group’s language difficulties were also a key factor for its comprehension performance to level to younger children with the same reading accuracy for text processing at its macro and super-structural levels.

These results confirm that LBLD suffer the effects of decoding deficits and it does not show the same compensation competence evidenced in the performance of children with dyslexia, once they present important deficits in linguistic abilities and essential competences for reading comprehension. These findings are possibly explained by language deficits, which prevent efficient activation of mechanisms implicated in reading.

Studies carried out with pupils with LBLD found less retold main ideas when compared with pupils of the same age and schooling (Williams, 1991; Curran et al., 1996; Carlisle, 1999; Puranik et al., 2008). Under control of the vocabulary variable, Carlisle (1999) also demonstrated that pupils with learning disabilities showed to be less able to understand and use textual structure as support to integrate the most important ideas of the text. Hansen (1978) points out that these pupils tended to present greater number of intrusions, in other words, they presented a greater number of pieces of information that did not belong to the text, a frequent behavior among children with important comprehension difficulties. Also, literature meta-analysis indicates that lots of studies report procedures that encourage pupils with learning disabilities to complement their retellings and, on the contrary, this strategy does not result in improvement of the total retold ideas or even the establishment of connections between the pieces of information (Reed and Vaughn, 2012). All of these findings suggest that the presence of language deficits restrains text processing at macro and super-structural levels, required for a global comprehension of what was read.

One of the explanations about comprehension difficulties presented by pupils with LBLD goes beyond decoding interference. It considers the influence of deficits in integrating information based on syntax problems, resulting from difficulties expressed throughout language development. Among the observed interferences, difficulties to accomplish inferences such as anaphora (Oakhill et al., 1986) and the worst performance of referential continuity in stories (Garnham et al., 1982) must also be highlighted. These manifestations would cause problems in understanding the role of linking elements and the organization of pieces of information – conjunctions and discourse markers – and would make the text processing at its macro and super-structural levels difficult, expressed by restricted number of retold links and by the worst reference standard of the retelling.

It is important to point out that, among limitations of the present, the low effect size found does not allow for the generalization of findings presented so far. New studies must be carried out in order to broaden evidences found in the present research.

## Conclusion

The reading comprehension assessment through a task of oral retelling after reading indicated that children with dyslexia and with LBLD showed difficulty in making sense of a read expository text. However, the groups presented impairments at different levels of text processing and different coverage of reading comprehension deficits.

Children diagnosed with developmental dyslexia showed more restricted impairments at macro-structural levels, considering the lower efficiency demonstrated to identify and choose main ideas when compared with typical pupils of the same age and schooling.

Children diagnosed with LBLD showed broader difficulties, impairing every level of text processing, in other words, micro, macro and super-structural levels.

The comparison of clinical groups with the performance of their typical pairs of the same reading accuracy also confirms the existence of differences in performance profile of dyslexic children and of children with LBLD. Such difference is possibly due to the distinct conditions of development of non-phonological dimensions of language observed in clinical groups.

Children diagnosed with developmental dyslexia showed better competence in retelling links between ideas present in the previously read text, and also achieved better retelling standards than pupils of the same decoding level. These findings suggest that the language competences and the knowledge acquired throughout schooling provide these children with better abilities to connect processed ideas at a macro-structural level, as well as to incorporate them into a broader textual scheme. Pupils with LBLD showed greater difficulty to connect ideas and also to build a global representation of the text.

At last, the different performance profiles in reading comprehension identified in the investigation of clinical groups with different types of Reading Disabilities suggest the possibility of achieving important indicators by means of the retelling after reading task. These indicators may help in a more precise diagnostic of reading comprehension impairment, making it more precise and specific. The study also shows the viability of using the retelling protocol as a means of accessing the mental elaboration built during reading, in which it is possible to determine the points of breakdown that may compromise reading comprehension. Its directive analysis may also be fundamental for clinical use, since, the precise identification of the comprehension level where difficulties remain allows for a more specific intervention upon these deficits. Besides, better understanding of difficulties may bring important educational developments as it enables the adoption of facilitating strategies and more assertive adaptations.

## Ethics Statement

This study was approved by the Research Ethics Committee of the Universidade Estadual Paulista “Júlio de Mesquita Filho” – UNESP – Marília (No. 0928/2014). The assessments started after the following: 1) authorization to collect data at the Clinic of Learning Disabilities at the Clinical Hospital of Faculdade de Medicina de Botucatu – Universidade Estadual Paulista “Júlio de Mesquita Filho” – UNESP – Botucatu and the Laboratory for Investigation of Learning Disabilities at the School of Philosophy and Sciences, Universidade Estadual Paulista “Júlio de Mesquita Filho” – CEES/FFC/UNESP – Marlia (SP).

## Author Contributions

AK prepared the project and the evaluation tool, collected and analyzed the statistics of the survey data, identified the literature, and wrote the article. CÁ collaborated in drawing up the assessment tool, in the discussion of survey data, and the preparation of the article. SC supervised the study, participated in the discussion of the data, and the preparation of the article.

## Conflict of Interest Statement

The authors declare that the research was conducted in the absence of any commercial or financial relationships that could be construed as a potential conflict of interest.
